# Effects of humic acid on enhanced removal of lead ions by polystyrene-supported nano-Fe (0) nanocomposite

**DOI:** 10.1038/s41598-020-76362-1

**Published:** 2020-11-12

**Authors:** Luyao Wang, Shiqiang Wei, Zhenmao Jiang

**Affiliations:** 1 Institute of Land Engineering and Technology, Shaanxi Provincial Land Engineering Construction Group Co., Ltd, Xi’an, 710075 China; 2 The Key Laboratory of Agricultural Resources and Environment in Chongqing, College of Resource and Environment, Southwest University, Chongqing, 400716 China

**Keywords:** Environmental sciences, Environmental chemistry, Nanoscale materials

## Abstract

Polymer-supported nanozero-valent iron composites (D001-nZVI) were fabricated for the removal of lead ions from aqueous solutions by embedding nZVI into the porous polystyrene anion exchanger D001. Humic acid (HA) was selected as a model species because of its ubiquitous existence to gain insight into the influencing factors in the actual application process. The iron contents of the composites were approximately 11.2%, and the smallest ZVI particle size was ~ 5 nm. The experimental results showed that the effect of HA on the reduction of lead ions by D001-nZVI was a concentration-dependent process. At low HA concentrations, the surface-competitive adsorption of HA and Pb^2+^ dominated; therefore, the removal efficiency of Pb^2+^ by D001-nZVI decreased from 97.5 to 90.2% with an increasing HA concentration. When the HA concentration increased to 30 mg/L or more, the lead ions removal remained constant with the following possible cooperation mechanism: the competitive adsorption of HA and Pb^2+^ on the nZVI surface and the well-dispersed particles were caused by electrostatic interactions between the HA coating and the nZVI surface. In addition, the adsorption complexation between HA and Pb^2+^ also had a positive effect on the removal of Pb^2+^ at higher concentrations of HA.

## Introduction

Heavy metal pollution in water is a global issue, such as the pollution by lead ions. The International Agency for Research on Cancer (IARC) has classified Pb as a possible carcinogen to humans (Group 2B). Although the quantity of Pb is only 0.0016% in the crust of the Earth^1^, serious Pb pollution mainly originates from industry, i.e., paint, battery, smelting, hardware, machinery, electroplating, cosmetics, hair dyes, glaze dishes. The Bulletin on Environmental Quality of China's Coastal Waters in 2015^2^ showed that the emission rate of lead ions was 0.3%, higher than the allowed limitation in China; in addition, civil health incidents caused by lead pollutants have also attracted widespread attention, such as serious blood-lead poisoning incidents in China and India^3^. Therefore, deep lead decontamination is urgent and significant. Conventional technologies include chemical precipitation, flocculation, ion exchange, adsorption, and membrane separation^4^. At present, chemical precipitation or flocculation is available for removing high concentrations of Pb ions, but it is inefficient at trace levels; reverse osmosis (RO) membranes are competent for removing trace heavy metals, including Pb ions, but their high operational cost significantly limits their applicability. Therefore, it is necessary to develop a cost-effective technology.

In recent years, transition metal oxides or M(0) and their composites have been industrialized as efficient adsorbents for heavy metal or diverse contaminant removal from water, such as ZrO_2_^5^, Fe(0)^6^ or Mn(IV)^7^. Since the 1990s, nanozero-valent iron (nZVI) has been widely used in water/soil pollution remediation due to its large specific surface area, strong reducing and adsorption-driven and environmentally friendly properties to remove contaminants^8,9^, such as NO_3_^–^N^8,10,11^, Cr(IV)^12^, Pb(II)^13,14^, As^15,16^ and organic pollutants^17,18^. Wang^19,20^ found that nZVI exhibited efficient Pb^2+^ removal in solution with efficiencies of ~ 99.8% at pH = 5, which resulted from the strong chemical reduction by nZVI and the adsorption by subsequent iron oxide cooperation on the surface of the nZVI particles.

However, nZVI tends to agglomerate into larger particles spontaneously to reduce its surface energy during its preparation and application^21^, thereby significantly reducing its active surface area and reactivity. To overcome these problems, nZVI was encapsulated onto a solid porous support or stabilized with a liquid dispersant to promote the dispersion of nZVI. Commonly used carriers are montmorillonite, bentonite^9,22^, activated carbon^23^, kaolinite^24^, resin^25,26^, etc., and liquid dispersants are mainly starch^27^, Tween-20, polyacrylic acid (PAA)^28^ etc. Our previous studies also showed that the microporous resin modified with a positively charged quaternary ammonium group was more favourable for forming smaller nZVI particles than most conventional carriers and led to a higher reduction activity for nitrate removal^29^. In addition, Chanthapon et al.^30^ also found that nZVI particles showed a well-dispersed morphology using cation exchange resin as a carrier, which further favoured Pb removal. The composite performance of organic carrier-inorganic nanoparticles is affected by the solution pH^14,31^, coexisting ions^32^, and the properties of natural humus in the reaction system in practical applications. However, studies on the effects of humic acid are still rare and insufficient^33^. Humic acid (HA) widely exists in natural water and industrial effluents, and it contains versatile functional groups, such as carboxylic acid, phenol hydroxyl, alcohol hydroxyl, ketone, quinone and ester groups^34^. There are three possible interactions between HA, heavy metal ions and nZVI. The first is a complexation between HA and the heavy metal ions. The abundant functional groups in HA can form a complex with heavy metal ions, in particular Pb^35^. In addition, the possible competitive adsorption between HA and Pb ions is also prevalent on the surface of iron oxides in nZVI. Wang and his coworkers^36^ also confirmed that the presence of competitive adsorption between HA and Cr(VI) led to a significant decrease in Cr(VI) removal when HA was added. Seunghun Kang^37^ also indicated that there were dual electrostatic interactions and complexation between the acidic functional groups of HA and the goethite surface. Liu et al.^38^ found that the adsorption of negatively charged HA on the surface of Fe_3_O_4_ resulted in overall negatively charged HA-Fe_3_O_4_, which effectively reduced the aggregation and further oxidation of Fe_3_O_4_ particles, thereby enhancing the adsorption of heavy metal ions, and the removal of Hg^2+^ and Pb^2+^ reached over 99%. Gupta and Nayak^39^ found that the effect of HA on the removal of Cd^2+^ by orange peel-Fe_3_O_4_ composites was related to the electrostatic attraction between the positively charged composites and negative-HA at pH < 7.

The current research mostly focuses on the effects of HA on the performance of bare nZVI materials, but immobilized nZVI, especially with cation exchange resin as supports, is rarely studied. HA shows different interactions with the resin carriers, nZVI, and heavy metals within nanopore environment of the matrix, supports, when polystyrene-based nZVI composites (denoted as D001-nZVI) are applied for Pb removal from water. To clarify the different mechanisms in this complex system, the interaction between HA and D001-nZVI, HA and Pb^2+^ was investigated first. Then, the direction and extent of the influence of HA on the removal performance of Pb^2+^ by D001-nZVI and the structure-related performance of D001-nZVI were analysed. In natural water, iron and nZVI-permeable reactive barriers frequently undergo long periods of HA adsorption and precipitation. Therefore, the present study aimed to evaluate nZVI performance in contaminated groundwater remediation under ambient conditions along with the investigation of the role of HA in the reduction of Pb(II) by nZVI particles. The results in this study may provide new insights into the application of nZVI composites in the detoxification of natural water.

## Materials and methods

### Materials

All chemicals used in this study, namely, FeCl_3_^.^6H_2_O, NaBH_4_, NaOH, PbCl_2_, and absolute ethyl ethanol, were all analytical reagent-grade or above. The support material is the macroporous cation exchanger D001, a polystyrene-divinylbenzene polymer matrix with sulfonic acid groups, purchased from Zhejiang Zhengguang Industrial Co., Ltd., China. Prior to use, it was rinsed with NaOH (5 wt%) and HCl (5 wt%), adjusted to a neutral pH with distilled water and dried at 40 °C for 24 h. All solutions were prepared with ultrapure water with a resistivity of 18.25 MΩ^.^cm. Humic acids were purchased from Tianjin Guangfu Technology Development Co., Ltd. and extracted from weathered coal. The HA sample was first dissolved in ultrapure water, and then the solution pH was adjusted to approximately 10 with 0.1 M NaOH. Afterward, the HA solution was ultrasonically treated for 2 h to fully dissolve it, and then the pH was adjusted to 7 with 0.1 M HCl. Finally, the HA solution was filtered through 0.45-μm membranes after being shocked for 24 h at room temperature. All prepared solutions were stored at 4 °C in the dark until further use^40^.

### Preparation of D001-nZVIs

Ultrapure water with a resistivity of 18.25 MΩ was purged with N_2_ gas purging for at least 1 h at room temperature and used for the preparation of nZVI to avoid oxidation during the modification process. Dry D001 resin beads (0.5 g) were added to 70 mL of a 2.0 M FeCl_3_ solution; after 12 h of rotation in an end-over-end shaker, the solid beads were obtained by filtration, rinsed five times with ultrapure water and then introduced into 70 mL NaBH_4_ solutions with different concentrations. After a certain time, the preloaded Fe^3+^ could be reduced in ZVI. The composites were freshly prepared before each experiment, and the materials used for structural characterization were dried in a vacuum oven at 40 °C for 24 h^40^.

### HA adsorption

D001 or the new synthesized D001-nZVI with the same amount of D001 was added into mixed solutions of different concentrations (0, 1, 2, 5, 10, 15, 20, 30, 50, and 100 mg/L) of HA and Pb^2+^ (500 mg/L), then shaken at a constant temperature of 298 K for 12 h at 120 rpm via a shaking-bed in a thermostatic water bath. The HA contents in the solutions before and after the reaction were measured^40^.

### Pb^2+^ removal

In all batch experiments, the dosages of the resin (D001) were set as 1.0 g/L. To investigate the performance of the lead ion removal by the nanocomposites, the beads were introduced into a 50 mL solution containing Pb^2+^ (500 mg/L) and different concentrations (0, 1.0, 2.0, 5.0, 10, 15, 20, 30, 50, 100 mg/L) of HA. The suspension was stirred at 120 rpm at a constant temperature of 298 K for 8 h. Then, the contents of Pb^2+^ and HA in the solution after the reaction were separately analysed^40^.

Kinetic tests were carried out to establish the effect of the contact time on the removal process and to quantify the removal rate. After adding the immobilized nZVI, 0.1000 g D001 was added to a flask containing 100 mL of a mixed solution of Pb^2+^ (500 mg/L) and different concentrations of HA (1, 5, 15, 40 mg/L), which was stirred at 298 K at a rate of 120 rpm. Moreover, the Pb^2+^ removal by the composites in the absence of HA was assessed as a control. Then, the concentration of residual Pb^2+^ in the solution was determined at regular time intervals by the methods described in the following section.

To monitor the concentration of Pb^2+^ over time, the new D001-nZVI composite (D001 dosage: 0.0500 g) was added to 50 mL of Pb^2+^ (500 mg/L) and different concentrations of HA (0, 1, 5, 15, 40 mg/L), and the reaction was terminated at 2, 5, 10, 15, 30, 45, 60, 90, 120, and 180 min, after which the D001-nZVI composites and the solution were immediately separated. The reaction solution was filtered through a 0.45 μm filter membrane to measure the concentration of Pb^2+^ in the filtrate (recorded as A), and the filter membrane and the precipitate were digested with nitric acid to determine the concentration of Pb^2+^ (recorded as B). After the reaction, the D001-nZVI composites were repeatedly eluted with 3 mol/L NaCl solution to detect the concentration of Pb^2+^ in the eluent (recorded as C), and the eluted particles were digested with nitric acid and the digested solution (recorded as D). A, B, C and D correspond to the amount of Pb^2+^ remaining in the solution after the reaction, the amount of precipitate formed by complexation of Pb^2+^ with HA, the amount of ion exchange of Pb^2+^ by carrier resin D001, and the amount of Pb^2+^ reduced by nZVI, respectively. The total Pb concentration is calculated as T_Pb_ = A + B + C + D.

### Characterization

Fourier transform infrared (FT-IR) spectra of HA were recorded at room temperature with a Spectrum GX spectrophotometer (Nicolet IS10). The microstructure and phase of the D001-nZVI composites before and after the reaction were measured by transmission electron microscopy (TEM, JEM 1200EX, Japan) and X-ray diffraction (XRD, Bruker D8). The cross-section of the D001-nZVI composites after vacuum-drying was observed by a bulk electron microscope (MicroDemo SZM45-B2).

### Analyses

The Pb^2+^ concentrations in the solutions were determined by a flame atomic absorption spectrophotometer (Beijing General Instrument Co., Ltd. TAS-900). Humic acid was determined spectrophotometrically by measuring the absorbance at λmax = 254 nm (Horiba JY Aqualog), and a standard curve was obtained to determine the residual concentration^40^. The XRD results were collected as binding energies and fitted using MDI Jade 6.0 software.

## Results and discussion

### Optimization of the preparation conditions of D001-nZVI

**Figure 1:**
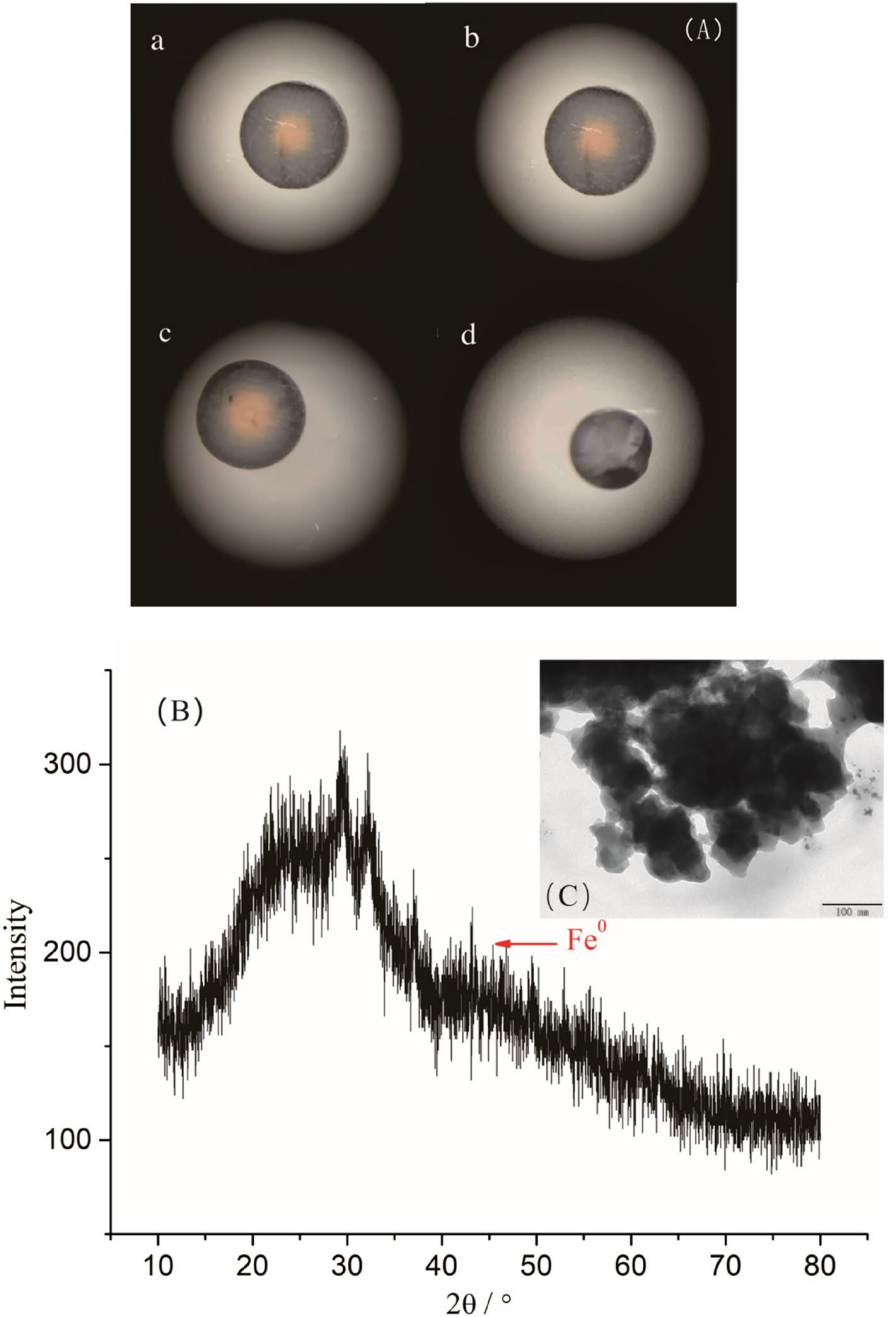
Micromorphology of D001-nZVI: (**A**) Micrograph of the D001-nZVI with different reducing agent concentrations and different reduction times; (**B**) TEM image of D001-nZVI; (**C**) XRD analysis of D001-nZVI.

The amounts of nZVI in D001-nZVI were closely related to the NaBH_4_ concentration and the reduction time, as shown in Table 1. The amounts of nZVI increased significantly when the concentration of NaBH_4_ and the reduction time were increased. The corresponding cross-sections of the D001-nZVI beads were also analysed by electron microscopy, and the results are shown in Fig. 1A. In the calculation from chemometrics, the Fe^3+^ adsorbed onto the matrix was completely reduced by the NaBH_4_ solution with a mass concentration of 0.05%. However, Fig. 1A shows that samples a, b and c still showed different shades of yellow (Fe^3+^), which indicated that the reduction of Fe(III) not only required a stoichiometric amount of reducing agent but also had a strong relationship with the diffusion of the reducing agent.

**Table 1:** The iron content of the resin in different reductant concentration and reduction time.

Samples	D001/g	FeCl_3_/M	NaBH_4_/%	Restore time/min	Average iron loading/%
a	0.05	2.0	3	15	8.98
b	0.05	2.0	3	30	9.91
c	0.05	2.0	4	15	9.50
d	0.05	2.0	4	30	11.20

The concentration gradient force was enhanced by increasing the reducing time and reductant concentration, which was beneficial for the complete reduction of Fe^3+^from the outside to the inside of the polymeric support bead. Therefore, the D001-nZVI used in the following experiments in this study was prepared by adding 7 mL of 2 mol/L FeCl_3_ per 0.05 g of D001, shaking for 12 h at 298 K and then carrying out its reduction by reacting with 7 mL of 4% NaBH_4_ solution for 30 min. The TEM image further shows the embedded nZVI morphology (Fig. 1B). The bulk aggregated background was ascribed to the polymeric matrix, and similar results were also observed in our previous study, while the embedded particles were assigned to nZVI with a size of ca. 5–10 nm (Fig. 1C). The XRD analysis of D001-nZVI (Fig. 1B) revealed that the characteristic peak of Fe(0) appeared at 44.9°^41^, which indicated the possible presence of Fe(0) nanoparticles inside the matrix.

### The reaction between HA and Pb^2+^ or D001-nZVI

**Figure 2:**
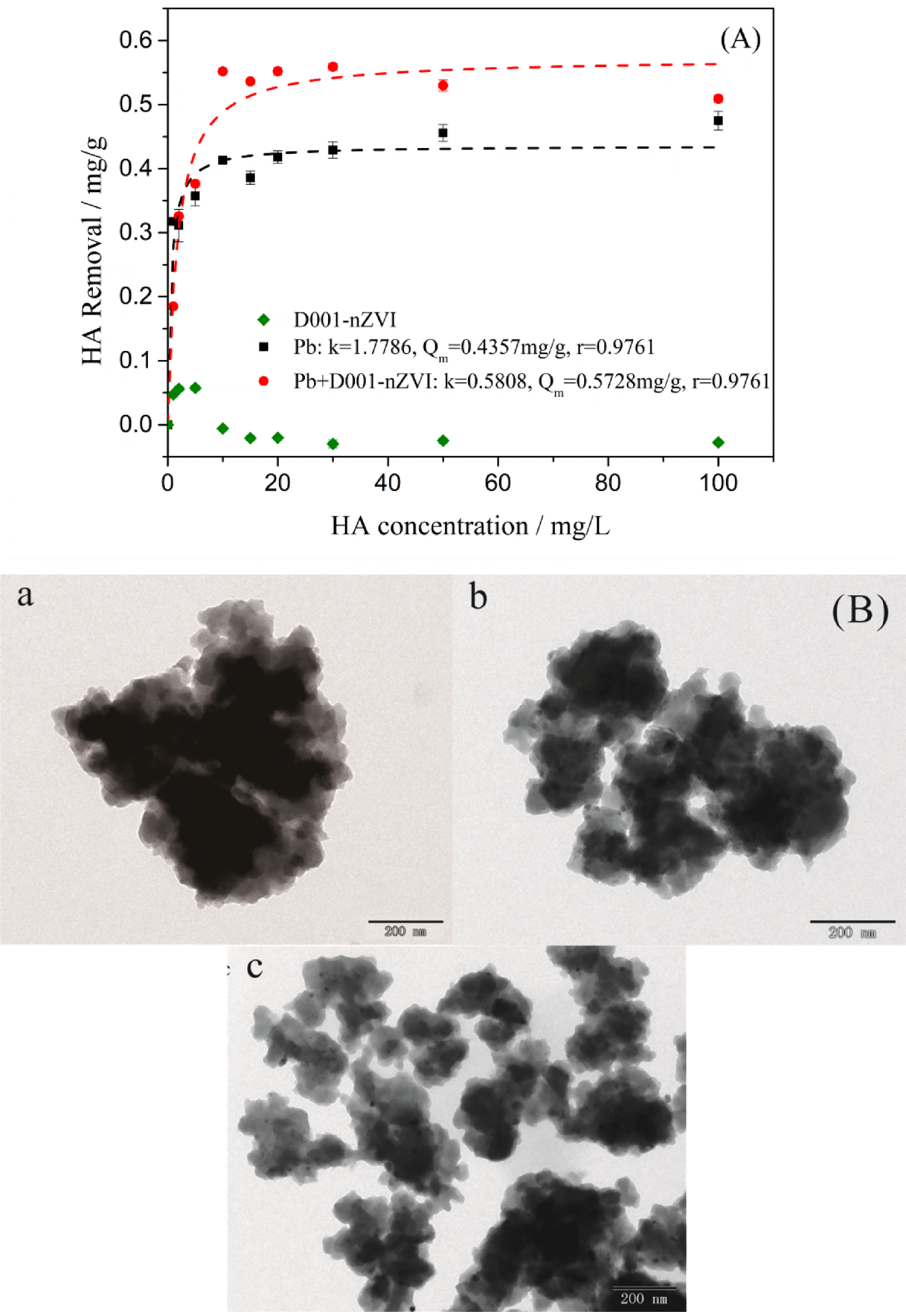
The adsorption characteristics of nZVI/ D001-nZVI and the TEM images before and after the reaction: (**A**) Adsorption isotherm of HA on D001, Pb^2+^, and the coexistence system of D001-nZVI and Pb^2+^; (**B**) TEM image of fresh D001-nZVI after reacted with (**a**) 0 mg/L, (**b**) 2 mg/L and (c) 100 mg/L HA (initial Pb^2+^, 500 ppm; T, 298 K; initial pH, 7; ZVI for all the reaction mixtures was the same and equal to 11.20 Fe% in mass).

**Figure 3:**
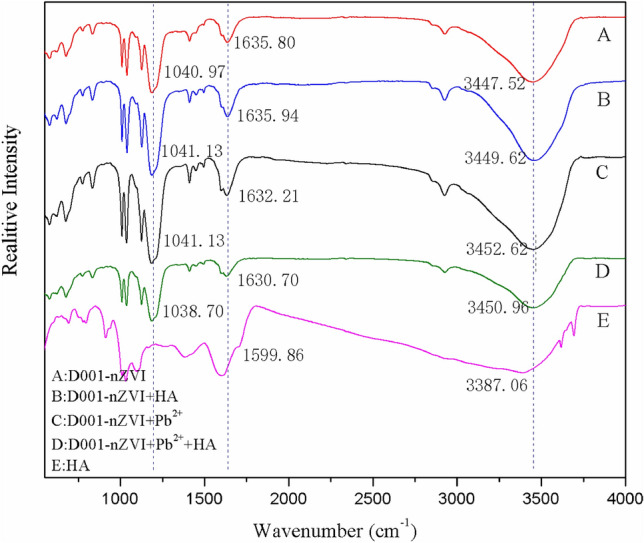
FT-IR spectra of (**A**) D001-nZVI; (**B**) D001-nZVI after reacted with 100 mg/L HA; (**C**) D001-nZVI after reacted with 500 ppm Pb^2+^; (**D**) D001-nZVI after reacted with 100 mg/L HA and 500 ppm Pb^2+^; (**E**) HA.

To further elucidate the interaction of the HA species with the hybrid D001-nZVI, a series of comparison experiments was conducted. Figure 2A shows the possible removal of HA by Pb^2+^ precipitation, D001-nZVI or binary D001-nZVI and Pb^2+^, respectively. Clearly, negligible HA adsorption onto D001-nZVI was observed, which was attributed to two different components, i.e., the matrix D001 and the embedded nZVI. Specifically, the negatively charged HA species exhibited strong electrostatic repulsion with the sulfonic acid group (-SO_3_H) of matrix D001. In addition, the adsorption of HA onto nZVI was also insignificant; the solution pH (pH = 5) was lower than the oxidation–reduction potential of iron oxide (pH = 7.9 ± 0.1), and the amount of iron oxides formed on the surface of nZVI was low, although the surface of iron oxide was positively charged^42^. This result was also confirmed by infrared spectroscopy analysis, as shown in Fig. 3. The major bands of HA appeared in the spectrum as follows: 3387.06 cm^-1^ (O–H stretching of hydroxyl), 1033.75 cm^-1^ (C–O stretching of primary alcohol), 1382.98 cm^-1^ and 1599.86 cm^-1^ (symmetric stretching of COO-, C–OH stretching of phenolic OH)^43^. Only the vibration of -OH (3447.52 cm^-1^) was slightly shifted to 3449.62 cm^-1^, which was attributed to the improved HA adsorption onto D001-nZVI.

Regarding the HA removal by Pb^2+^ precipitation, the HA species readily formed complex precipitates with Pb^2+^, and both Pb^2+^ and HA could be removed from the solution as precipitates. It is noteworthy that HA removal remained constant when the added HA concentration was 10 mg/L or higher, which was ascribed to the complete complexation of Pb ions. Interestingly, the binary D001-nZVI and Pb^2+^ systems exhibited favourable HA removal, indicating that HA can not only be complexed with Pb^2+^ as precipitates but also be adsorbed by iron oxides on the outer surface of nZVI. The process was accompanied by the continuous formation of iron oxide, which occurred along with the Pb^2+^ reduction by nZVI and further improved the adsorption of HA. Thus, the removal of HA by binary D001-nZVI and Pb^2+^ was more efficient. In addition, HA may interact with the exterior iron oxide of the nZVI shell, upon which the negative charge of HA can neutralize the positively charged iron oxide and generate a negatively charged nZVI surface by forming an organic macromolecular coating (hereinafter referred to as HA coating). The aggregation of the nZVI particles was inhibited by the electrostatic repulsion of the HA coating, which further increased the reactivity of nZVI^44^. The FTIR analysis of D001-nZVI after reacting with Pb^2+^ before and after adding HA also confirmed this result. As shown in Fig. 3, the vibration of –SO_3_H (1041.13 cm^−1^), the vibration of benzene (1632.21 cm^−1^) and the vibration of –OH (3452.62 cm^−1^) in D001-nZVI were shifted to 1038.70 cm^−1^, 1630.70 cm^−1^, and 3450.96 cm^−1^, respectively, after HA was added, which proved that HA promoted the adsorption of D001-nZVI.

To further investigate the possible sorption mechanism, TEM images of D001-nZVI before and after reaction with HA at low (2 mg/L) and high (100 mg/L) concentration are shown in Fig. 2B. It was found that the addition of HA resulted in nZVI particles with a well-dispersed morphology, and similar results were also obtained in a previous study^25^.

The formed HA coating on nZVI was negatively charged, which generated electrostatic repulsion, resulting in well-dispersed nZVI particles. In contrast, when the concentration of HA was low, charge neutralization between HA and the iron oxide surface may cause nZVI particle aggregation, while at high concentrations of added HA, the present steric hindrance and electrostatic repulsion significantly inhibited the aggregation of the particles^44^. Overall, three mechanisms for the HA and Pb^2+^ interaction were found, i.e., HA and Pb^2+^. The complexation of HA and Pb^2+^ as precipitates increased the nZVI reactivity through electrostatic repulsion due to the HA coating and possible adsorption competition between HA and Pb^2+^ on the iron oxide interface. The first two approaches favoured lead ion removal, while the last approach possibly inhibited the Pb^2+^ adsorption.

### Effect of HA on the removal efficiency of Pb^2+^ by D001-nZVI

**Figure 4:**
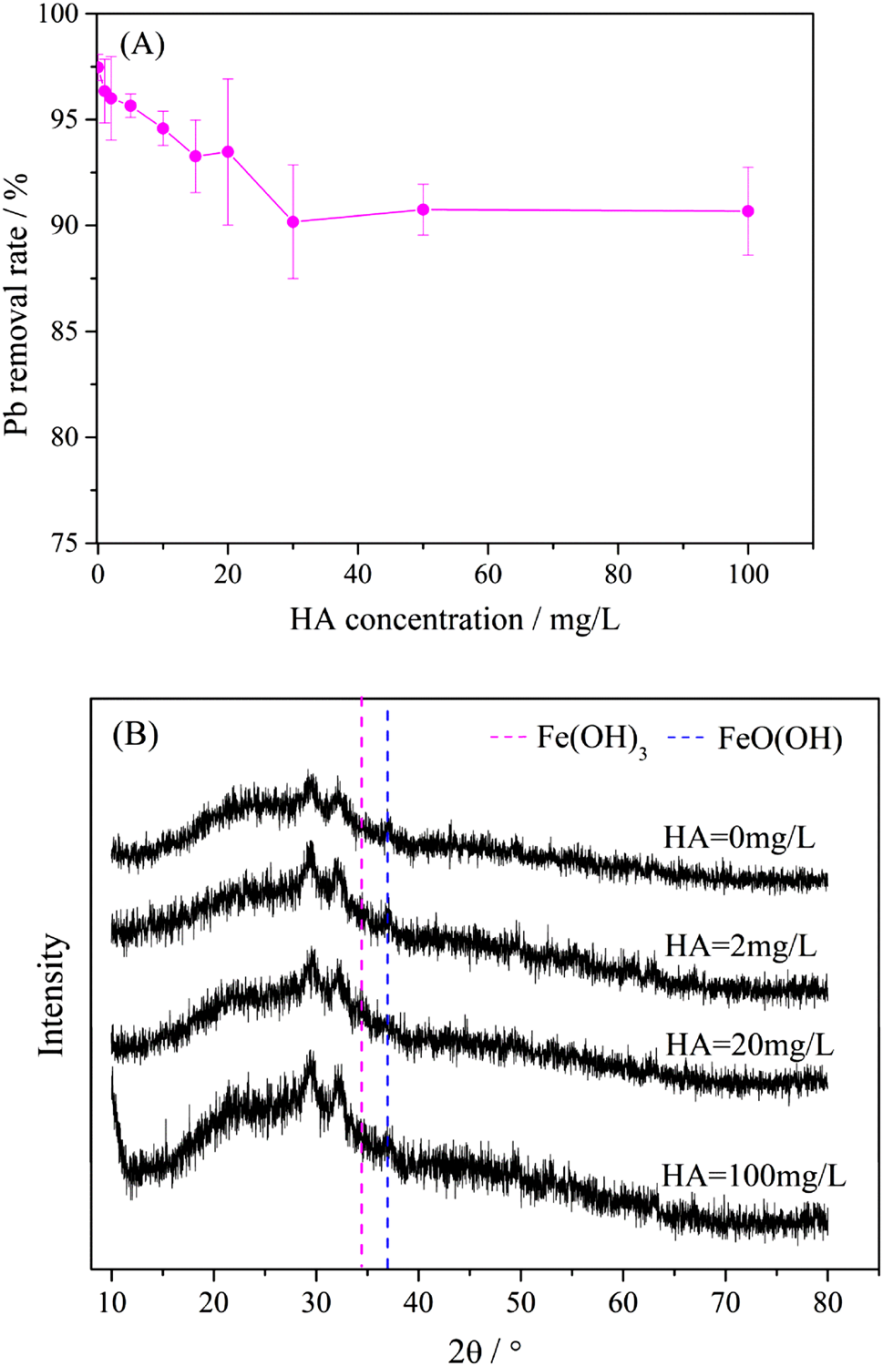
nZVI removal characteristics of Pb and changes of its chemical composition: (**A**) Removal rate of lead ions in different concentration of initial HA; (**B**) XRD pattern of D001-nZVI reacted with Pb^2+^ under different concentrations of HA (initial Pb^2+^, 500 ppm; T, 298 K; initial pH, 7; ZVI for all the reaction mixtures was the same and equal to 11.20 Fe% in mass).

Figure 4A shows the effects of different concentrations of HA on the removal of Pb^2+^ by D001-nZVI. The removal efficiency of Pb^2+^ was 97.41% in the absence of HA and gradually decreased with increasing HA addition. In particular, the removal of Pb^2+^ was approximately 90% and remained constant when HA > 30 mg/L. Based on the possible removal mechanisms mentioned, the competitive adsorption of HA and Pb^2+^ dominated the sorption process, leading to a decrease in Pb^2+^ removal. The XRD patterns of D001-nZVI showed different changes in the characteristic diffraction peaks after reacting with Pb^2+^ with HA backgrounds (Fig. 4B). The diffraction peak at 2θ of 29.6° corresponded to the amorphous Pb(OH)_2_, crystal PbO^.^xH2O morphology corresponding to 2θ at 32.2° and Pb(0), corresponding to 2θ of 55.4° and 62.4^23,45^, which indicated that nZVI could reduce Pb(II) to Pb(0).

**Figure 5:**
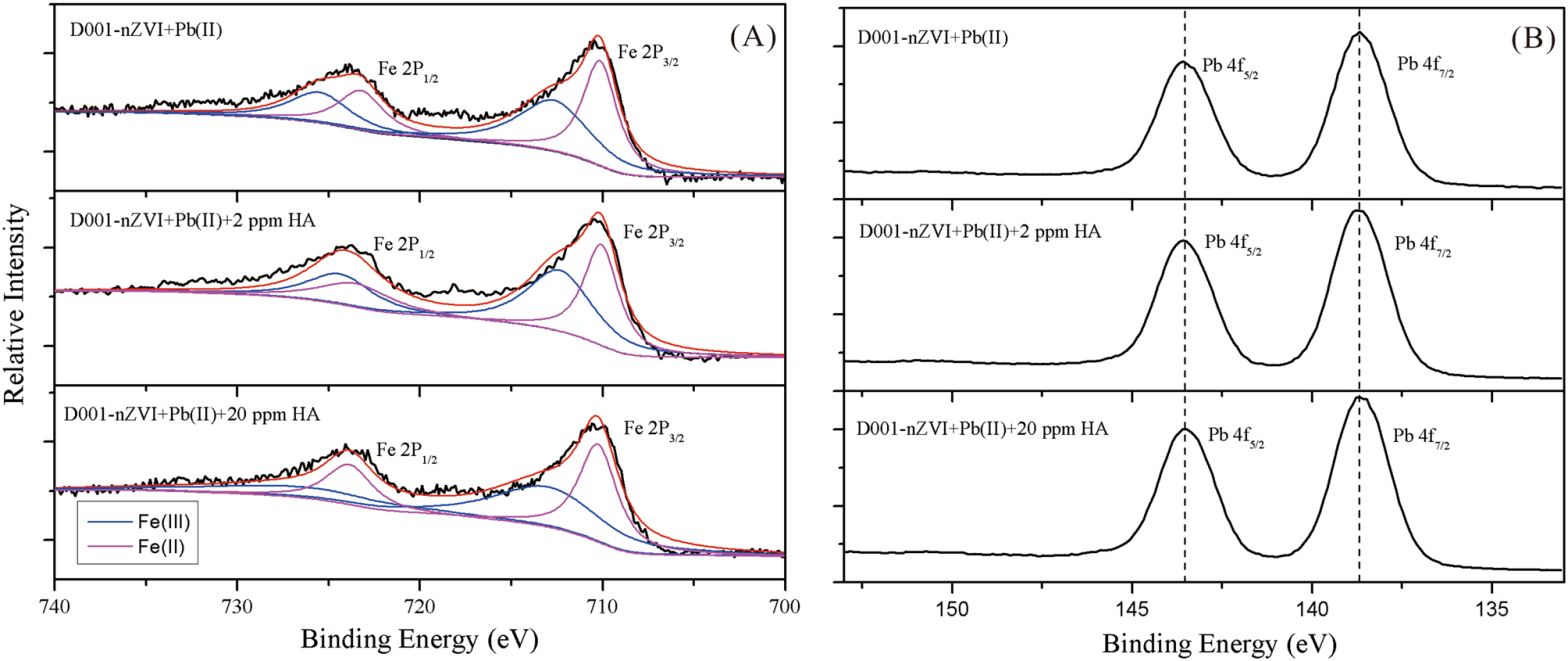
XPS spectra for Fe (**A**) and Pb (**B**) on the D001-nZVI after reacted with Pb^2+^ under different concentration of HA (initial Pb^2+^, 500 ppm; T, 298 K; initial pH, 7; HA concentration are 0 ppm, 2 ppm, 20 ppm, respectively).

The standard reduction potential of Pb(II)/Pb(0) is − 0.1263 V, which is higher than the standard reduction potential of Fe(0)/Fe(II) (− 0.4402 V)^46^; thus, Pb(II) can easily be reduced by nZVI. Moreover, the iron oxide content and the pH of the solution (Figure S1) also increased slightly during the oxidation of Fe(0) to Fe(II) or/and Fe(III), which was ascribed to the oxidation and hydration of Pb(0) on the surface of D001-nZVI^45^. However, these Fe(0) (2θ = 44.9°) peaks were weakened or disappeared completely in D001-nZVI (Fig. 4B), and the emerging peaks at 34.9° and 36.6° proved the presence of Fe(OH)_3_ and FeOOH species, respectively. In addition, a strong peak attributed to FeOOH species was detected; when the HA concentration was lower than 2 mg/L, a further increase in the HA concentration led to the transformation of the FeOOH to the Fe(OH)_3_ species. Similar results were also found in a recent study, in which the presence of Fe(II) ions could promote the conversion of ferrihydrite to FeOOH at room temperature and neutral pH^47^. Therefore, when the concentration of HA was low (< 2 mg/L), the reduction of Pb^2+^ was accompanied by the generation of Fe^2+^, and high amounts of FeOOH were obtained during this stage; the opposite was the case for Fe(OH)_3_. This was also confirmed by XPS analysis, as shown in Fig. 5. The content of Fe(II) on D001-nZVI after reacting with Pb^2+^ at concentration lower than 2 ppm of HA was reduced, which proved that more Fe^2+^ was involved in the reaction. When the concentration of HA was continuously increased (> 2 mg/L), the surface of nZVI particles continuously adsorbed HA to form the HA coating. As shown in Fig. 5, the content of Fe(II) on D001-nZVI after reacting with Pb^2+^ at concentrations lower than 20 ppm HA remained almost unchanged compared with 0 ppm HA and increased compared with 2 ppm HA. It has been found that some ions or molecules can adsorb on the surface of Fe(OH)_3_ to inhibit the formation of FeOOH, such as silicate, phosphate, citrate ions and sodium dodecyl sulfate (SDS)^48^. Thus, we concluded that the HA coating on the nZVI surface inhibited the formation of FeOOH, which resulted in a decrease in its content. A previous study also showed that FeOOH can adsorb heavy metal ions^49^, which is beneficial to the removal of heavy metal ions; thus, the decrease in FeOOH content may also be one of the reasons for the decrease in the Pb^2+^ removal.

### Effect of HA on the Pb^2+^ removal kinetics

**Figure 6:**
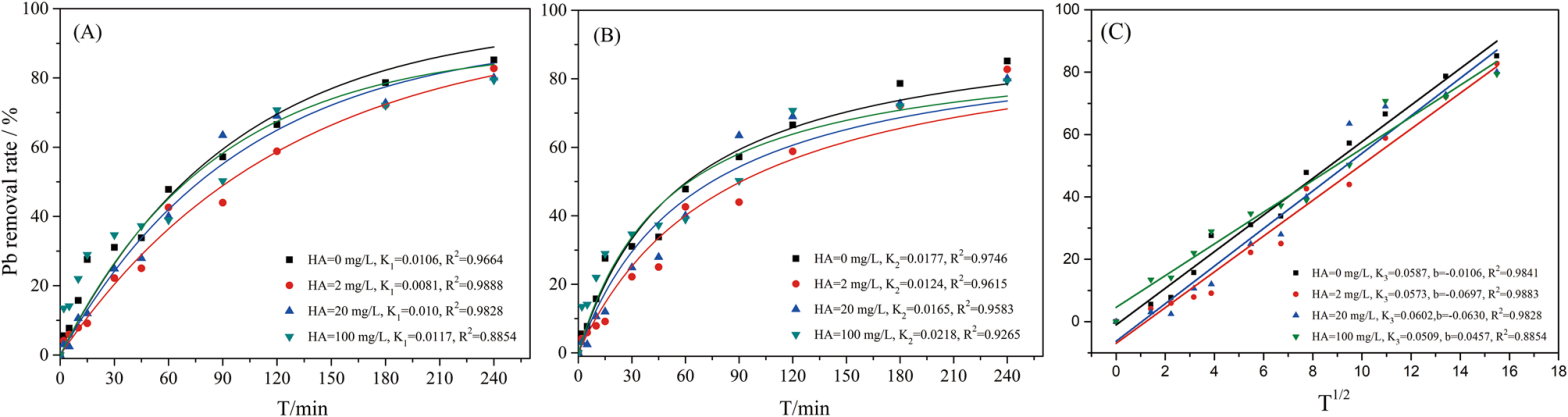
The kinetic characteristics of Pb^2+^ removal by D001-nZVI at different concentration of HA: (**A**) the first-order kinetic model curve; (**B**) the second-order kinetic model curve; (**C**) Webber-Morris kinetic model curve.

Kinetic performance is one of the important indicators for evaluating the efficiency of the adsorbent. As shown in Fig. 6, sorption equilibrium was completed in ~ 200 min, and HA addition at different concentrations showed similar sorption behaviours. The relationship between the Pb^2+^ removal rate and the removal time at different HA concentrations was fitted by the first-order kinetic model and the second-order kinetic model, which can be expressed as follows:

$$ \begin{aligned} \left[ C \right]_{t} & = \left[ C \right]_{e} \left( {1 - e^{{ - k_{1} t}} } \right) \\ \frac{t}{{\left[ C \right]_{t} }} & = \frac{1}{{k_{2} \left[ C \right]_{e}^{2} }} + \frac{t}{{\left[ C \right]_{e} }} \\ \end{aligned} $$

where k_1_ and k_2_ (min^−1^) represent the first-order kinetic reaction constant and the second-order kinetic reaction constant, respectively, and [C]t represents the removal rate of Pb^2+^ in the solution at time t. The fitting parameters of the results are shown in Table S1. The results could be described well by the second-order kinetic model with higher R^2^ values, and the [C]_e,cal2_ was also closer to [C]_e,exp_ than to [C]_e_,_cal1_. The value of k_2_ decreased when the HA concentration increased from 0 to 2 mg/L and may increase to 0.022 when HA > 2 mg/L, which is higher than the value of k_2_ without HA.

However, the removal rate of Pb^2+^ in the solution conforms to the first-order kinetic model and the second-order kinetic model, and the rate control step cannot be determined. In general, the rate of adsorption kinetics usually depends on three main steps, including external diffusion, intraparticle diffusion, and interaction between the adsorbate and the adsorbents^50^. To determine the actual control steps of the adsorption rate, the results were also analysed by Webber-Morris (intraparticle diffusion model), which describes the overall intraparticle diffusion effect^51,52^. The Webber-Morris (intraparticle diffusion model) can be expressed as follows:

$$ \left[ C \right]_{t} = k_{3} t^{1/2} + b $$

where k_3_ (min^1/2^) represents the Webber-Morris kinetic reaction constant and b represents the boundary layer index. As shown in Fig. 6C, when the concentration of HA was 0 ~ 20 mg/L, the removal rate of Pb^2+^ was well-fitted by the intraparticle diffusion model, and the correlation coefficients were 0.9828 ~ 0.9883. This result showed that the intraparticle diffusion in the removal process of D001-nZVI to Pb^2+^ was the rate-limiting step at concentrations of 0 ~ 20 mg/L HA. The b values of the removal of Pb^2+^ by D001-nZVI were -0.0697 ~ 0.0457, which were all greater than 0. This result indicated that intraparticle diffusion was not the only rate-limiting process. Pb^2+^ diffusion through the membrane to reach the surface of D001-nZVI also affected the removal.

The concentrations of Pb^2+^ in the solution decreased at low HA concentrations (2 ~ 20 mg/L) due to the complexation of HA and Pb^2+^, indicating that the concentration of Pb^2+^ in the bulk solution decreased, which also led to a dramatic weakening of the concentration driving force. Thus, the external diffusion rate declines. Moreover, the thickness of the particle boundary layer increased due to the HA coating, which also largely limited the internal diffusion of the particles. When the concentration of HA was higher than 20 mg/L, the complexation of HA and Pb^2+^ was no longer dominant; at this time, Pb^2+^ diffusion through the membrane to reach the surface of D001-nZVI controlled the sorption process.

### The fate of Pb^2+^ after reaction with D001-nZVI

**Figure 7:**
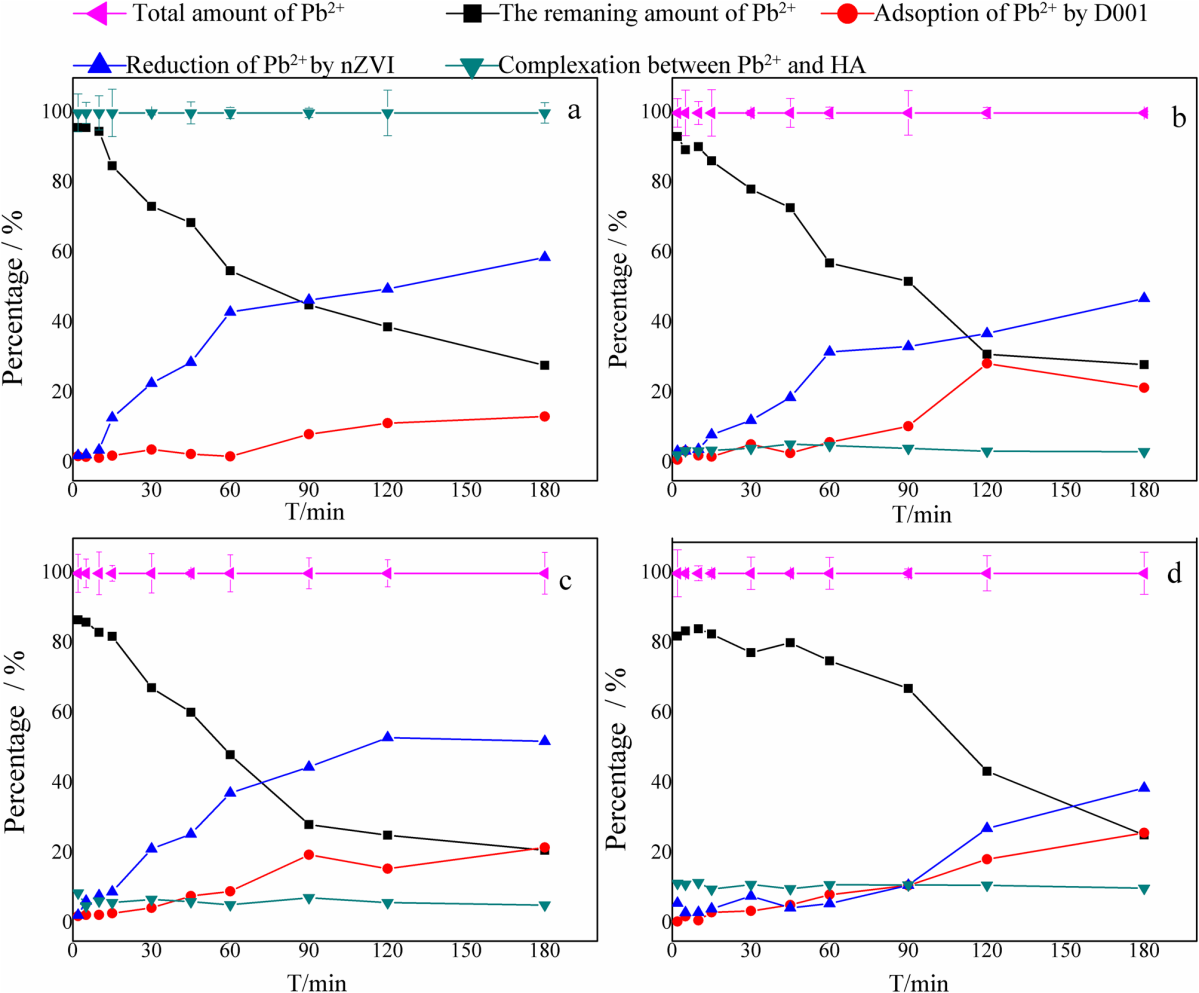
The removal of Pb^2+^ by D001-nZVI at different time in different concentration of HA (Figure **a**, **b**, **c**, **d** correspond to HA concentration of 0 mg/L, 2 mg/L, 20 mg/L, 100 mg/L).

The removal pathway of Pb^2+^ by D001-nZVI in this experimental system included (1) adsorption of Pb^2+^ by the matrix resin D001; (2) reduction of Pb^2+^ by nZVI; and (3) complexation between HA and Pb^2+^. Therefore, it is necessary to explore the main removal pathway of Pb^2+^ as well as the sorption-reduction mechanism at different concentrations of HA. Figure 7 shows an investigation of the Pb mass balance in the reaction system. It was found that the total Pb mass in the system remained at the same level within the error bars and could be correlated with the amount of reduction of Pb^2+^ by nZVI, adsorption of Pb^2+^ by the carrier resin, complexation and the amount of remaining Pb species; the detailed proportions at different concentrations are shown in Fig. 7. The reaction strength order was as follows: reduction of Pb^2+^ by nZVI > adsorption of Pb^2+^ by D001 > complexation of HA and Pb^2+^.

The amount of complex precipitation of Pb^2+^ and HA did not change significantly over time, regardless of whether HA was added. The amount of Pb^2+^ complexation was lower than 0.1% without HA, and when HA = 2, 20 and 100 mg/L, the average amounts of complexation between HA and Pb^2+^ were 3.87%, 6.34% and 10.85%, respectively, and increased with increasing HA concentration. This process promoted the removal of Pb^2+^ to a certain extent.

Overall, the proportion of this fraction was at least twice the amount of adsorption by D001, which indicated that the reduction of Pb^2+^ by nZVI played a major role in the removal of Pb^2+^ by D001-nZVI. Some of the Pb^2+^ removed by nZVI increased over time when HA was added. At the same time points, the amount of removal was 0 mg/L HA > 20 mg/L HA > 2 mg/L HA > 100 mg/L HA. There was a competition between HA and Pb^2+^ for active sites on nZVI, although the HA coating on the nZVI surface results in a better dispersion of the nZVI particles. Therefore, the proportion of Pb^2+^ removed by nZVI decreased with increasing HA. The XRD analysis of Fig. 4B showed that the characteristic peak intensity of FeOOH increased with increasing HA concentration when the HA concentration was lower than 20 mg/L, and FeOOH could adsorb heavy metal ions. Therefore, when the HA concentration was 20 mg/L, the reduction was more efficient than that at HA concentrations of 2 mg/L and 100 mg/L. Moreover, the adsorption amount of Pb^2+^ increased over time after adding different concentrations of HA, and in general, the amount of Pb^2+^ adsorption increased with increasing HA concentration, which indicated that the addition of HA promoted the adsorption of Pb^2+^ on D001. When nZVI was loaded on D001, the positive charge of the oxidation shell surface on nZVI slightly reduced the overall electronegativity of D001-nZVI. After adding HA, the electrostatic attraction between HA and the nZVI oxide shell neutralized the positive charge and even generated an overall negatively charged surface; thus, the overall electronegativity of D001-nZVI increased, which increased the adsorption of Pb(II).

## Conclusions

In summary, a new nanocomposite, D001-nZVI, was prepared through ion exchange and chemical reduction, and investigated in terms of the HA effects on the removal of Pb^2+^. The result shows that the performance of HA on D001-nZVI removal of Pb^2+^ was related to its concentration. The Pb^2+^ removal rate decreased as the HA concentration increased, but when the HA concentration was greater than 30 mg/L, the Pb^2+^ removal rate remained constant. There were three main mechanisms influencing the reaction process: (1) the complexation between HA and Pb^2+^, (2) the formation of an HA coating due to the adsorption of HA on the surface of nZVI, and (3) competitive adsorption between HA and Pb^2+^ on the iron oxide on the surface of nZVI particles. The former mechanisms two can facilitate the D001-nZVI removal performance, but their effects were all less pronounced than the negative effects caused by the third mechanism. In addition, the adsorption complexation between HA and Pb^2+^ also played a positive role in the removal of Pb^2+^ at higher concentrations of HA. Therefore, in practice, it is necessary to consider the concentration of HA present in the water when nanocomposites are used to remove heavy metal lead ions.

## Supplementary information

Supplementary Information.
