# Supplementary material for: Effects of humic acid on enhanced removal of lead ions by polystyrene-supported nano-Fe (0) nanocomposite
Source: Sci Rep. 2020 Nov 12;10:19663. doi: 10.1038/s41598-020-76362-1 (PMC7665180; doi:10.1038/s41598-020-76362-1)
Supplement: Supplementary file 1 — Supplementary Information. [file 41598_2020_76362_MOESM1_ESM.docx]

**Effects of humic acid on enhanced removal of lead ions by polystyrene-supported nano-Fe (0) nanocomposite**

Luyao Wang ^a, b^, Shiqiang Wei ^b^, Zhenmao Jiang ^b^*

^a^ Institute of Land Engineering and Technology, Shaanxi Provincial Land Engineering Construction Group Co., Ltd., Xi’an 710075, China.

^b^ The key laboratory of agricultural resources and environment in Chongqing, College of resource and environment, Southwest University, Chongqing 400716, China

*To whom correspondence should be addressed

E-mail: [windring@swu.edu.cn](mailto:windring@swu.edu.cn) (Z. Jiang)

Tel: +86-23-6825-1249

Supplementary material

**Fig.S1** Solution pH of after between D001-nZVI and Pb^2+^ under different concentrations of HA





**Table S1**

Pseudo-first-order, Pseudo-second-order and Webber-Morris kinetic constants of lead ions removal rate in different concentration of HA

| HA/ mg/L | [C]_e,exp_ | Pseudo-first-order kinetics | | | Pseudo-second-order kinetics | | | Webber-Morris kinetics | | |
| --- | --- | --- | --- | --- | --- | --- | --- | --- | --- | --- |
|  |  | [C]_e,cal1_ | k_1_ | R^2^ | [C]_e,cal2_ | k_2_ | R^2^ | K_3_ | b | R^3^ |
| 0 | 0.975 | 0.964 | 0.011 | 0.966 | 0.976 | 0.018 | 0.974 | 0.059 | -0.011 | 0.984 |
| 2 | 0.960 | 0.940 | 0.008 | 0.989 | 0.961 | 0.012 | 0.968 | 0.057 | -0.070 | 0.988 |
| 20 | 0.935 | 0.924 | 0.010 | 0.983 | 0.935 | 0.016 | 0.953 | 0.060 | -0.063 | 0.983 |
| 100 | 0.907 | 0.891 | 0.012 | 0.885 | 0.907 | 0.022 | 0.905 | 0.051 | 0.046 | 0.885 |
